# Chloroplast DNA rearrangements in Campanulaceae: phylogenetic utility of highly rearranged genomes

**DOI:** 10.1186/1471-2148-4-27

**Published:** 2004-08-23

**Authors:** Mary E Cosner, Linda A Raubeson, Robert K Jansen

**Affiliations:** 1 (Deceased) Department of Plant Biology, Ohio State University, Columbus, OH 43210 USA; 2 Department of Biological Sciences, Central Washington University, Ellensburg, WA 98926-7537, USA; 3 Section of Integrative Biology and Institute of Cellular and Molecular Biology, University of Texas, Austin, TX 78712 USA

## Abstract

**Background:**

The Campanulaceae (the "hare bell" or "bellflower" family) is a derived angiosperm family comprised of about 600 species treated in 35 to 55 genera. Taxonomic treatments vary widely and little phylogenetic work has been done in the family. Gene order in the chloroplast genome usually varies little among vascular plants. However, chloroplast genomes of Campanulaceae represent an exception and phylogenetic analyses solely based on chloroplast rearrangement characters support a reasonably well-resolved tree.

**Results:**

Chloroplast DNA physical maps were constructed for eighteen representatives of the family. So many gene order changes have occurred among the genomes that characterizing individual mutational events was not always possible. Therefore, we examined different, novel scoring methods to prepare data matrices for cladistic analysis. These approaches yielded largely congruent results but varied in amounts of resolution and homoplasy. The strongly supported nodes were common to all gene order analyses as well as to parallel analyses based on ITS and *rbcL *sequence data. The results suggest some interesting and unexpected intrafamilial relationships. For example fifteen of the taxa form a derived clade; whereas the remaining three taxa – *Platycodon*, *Codonopsis*, and *Cyananthus *– form the basal clade. This major subdivision of the family corresponds to the distribution of pollen morphology characteristics but is not compatible with previous taxonomic treatments.

**Conclusions:**

Our use of gene order data in the Campanulaceae provides the most highly resolved phylogeny as yet developed for a plant family using only cpDNA rearrangements. The gene order data showed markedly less homoplasy than sequence data for the same taxa but did not resolve quite as many nodes. The rearrangement characters, though relatively few in number, support robust and meaningful phylogenetic hypotheses and provide new insights into evolutionary relationships within the Campanulaceae.

## Background

The Campanulaceae *sensu stricto *are a nearly cosmopolitan angiosperm family consisting of latex-bearing, primarily perennial herbs or occasional subshrubs that typically have alternate leaves, sympetalous corollas, inferior ovaries, and capsular fruits. Allied to the Campanulaceae are the Lobeliaceae, Cyphiaceae, Cyphocarpaceae, Nemacladaceae, Pentaphragmataceae, and Sphenocleaceae; at times, all of these taxa have been included in the Campanulaceae at varying taxonomic rank by different authors (Table 1). Taxonomic treatments lack consensus (Table 1) and phylogenetic work has only recently been attempted. Campanulaceae in the strict sense are recognized as 600 [1] to 950 [2] species distributed among 35 [1] to 55 [2] genera. Generic circumscription and intrafamilial classification vary widely according to author. Within the family as few as two [3] and as many as 18 [4] tribes have been recognized (Table 1). Fedorov's more recent work [5] recognized eight tribes (Table 1), but only included taxa present in the former Soviet Union. Although Kolakovsky's treatment of Old World Campanulaceae [4] is the most recently published attempt to produce a more complete intrafamilial classification of the Campanulaceae (Table 1), the scope of the work is limited compared to that of either A. de Candolle [3] or Fedorov [5]. In all treatments, the Campanuleae and Wahlenbergieae (at whatever rank) are typically the largest, most inclusive taxa, with segregate tribes consisting of only one to a few genera.

**Table 1:** Classification systems of Campanulaceae. All major intrafamilial subdivisions are included (level of subdivisions indicated by number of dashes) but only those genera sampled in this study are included. If sampled genera are not listed, the genus was not recognized by the author but, rather, was subsumed into one of the listed genera.

A deCandolle	AP deCandolle	Schönland	Federov	Takhtajan	Kovanda	Kolakovsky
1830 [3]	1839 [53]	1889 [45]	1972 [5]	1987 [2]	1978 [1]	1987 [4]
**Campanulaceae**	**Campanulaceae**	**Campanulaceae**	**Campanulaceae**	**Campanulaceae**	**Campanulaceae**	**Campanulaceae**
**-Subtribus I**	**-Wahlenbergieae**	-**Lobelioideae**	**-Sphenocleoideae**	-**Cyanantheae**	-**Campanulinae**	-**Prismatocarpoideae**
*Jasione*	*Jasione*	**-Cyphioideae**	**-Campanuloideae**	*Cyananthus*	*Campanula*	*Prismatocarpus*
*Codonopsis*	*Platycodon*	**-Campanuloideae**	--**Campanuleae**	**-Wahlenbergieae**	*Symphyandra*	*Roella*
*Platycodon*	*Codonopsis*	**--Pentaphragmeae**	*Campanula*	*Wahlenbergia*	*Legousia*	-**Canarinoideae**
*Wahlenbergia*	*Wahlenbergia*	**--Sphenocleae**	*Symphyandra*	*Edraianthus*	-**Wahlenberginae**	-**Wahlenbergoideae**
*Prismatocarpus*	*Prismatocarpus*	**--Campanuleae**	**--Peracarpeae**	*Jasione*	*Wahlenbergia*	--**Wahlenbergieae**
*Roella*	*Roella*	**Campanulinae**	**--Ostrowskieae**	*Codonopsis*	*Codonopsis*	*Jasione*
**-Subtribus II**	*Edraianthus*	*Symphyandra*	**--Michauxieae**	*Merciera*	*Cyananthus*	*Wahlenbergia*
*Petromarula*	**-Campanuleae**	*Trachelium*	**--Phyteumateae**	*Roella*	*Roella*	*Codonopsis*
*Campanula*	*Petromarula*	*Campanula*	*Asyneuma*	*Prismatocarpus*	*Edraianthus*	*Platycodon*
*Trachelium*	*Campanula*	**Wahlenberginae**	*Legousia*	**-Platycodoneae**	*Jasione*	*Cyananthus*
*Symphyandra*	*Trachelium*	*Cyananthus*	--**Wahlenbergieae**	*Platycodon*	**-Platycodinae**	**--Azorineae**
*Musschia*	*Symphyandra*	*Jasione*	*Codonopsis*	*Musschia*	*Platycodon*	--**Musschieae**
**-incertae sedis**	*Musschia*	*Prismatocarpus*	**--Edraiantheae**	-**Campanuleae**		*Musschia*
*Merciera*	**-Merciereae**	*Merciera*	*Edraianthus*	*Campanula*		--**Echinocodoneae**
	*Merciera*	*Edraianthus*	--**Jasioneae**	*Legousia*		--**Annaea**
		*Wahlenbergia*	*Jasione*	*Triodanis*		**--Muehlbergelleae**
		*Codonopsis*		-**Michauxieae**		**--Theodorovieae**
		*Roella*		**-Phyteumaeae**		**--Gadellieae**
		**Platycodinae**		*Asyneuma*		**--Ostrowskieae**
		*Platycodon*		*Trachelium*		-**Campanuloideae**
		*Musschia*		*Petromarula*		--**Campanuleae**
				**-Peracarpeae**		*Campanula*
						*Symphyandra*
						*Trachelium*
						**--Phyteumateae**
						**--Peracarpeae**
						**--Sergieae**
						**--Michauxieae**
						--**Neocodoneae**
						*Asyneuma*
						*Legousia*
						--**Edraiantheae**
						*Edraianthus*
						**--Sachokieleae**
						**--Mzymteleae**

The most comprehensive treatment of the Campanulaceae remains the monograph of A. de Candolle [3], who recognized two groups corresponding to the Wahlenbergieae and Campanuleae (Table 1). Simple basal leaves and simple, alternate or occasional whorled, cauline leaves that are often different in shape than the basal leaves, characterize the Campanuleae in de Candolle's sense. Flowers are solitary or borne in cymes or racemes, and have five corolla lobes that are mostly fused proximally. The inferior ovary usually has 3–5 carpels and develops into a capsule that mostly dehisces by lateral pores (rarely a berry). The Wahlenbergieae are mostly perennials characterized by simple, alternate, cauline leaves. Flowers are solitary or borne in cymes or heads, and petals may be free, proximally fused, or distally fused. The ovary is inferior, semi-inferior, or superior, and consists of two, three, or five carpels. The fruit is generally a capsule dehiscing by apical pores or valves (rarely a berry). Both groups have five stamens with filaments that are often proximally dilated and anthers with introrse dehiscence; nectaries are generally present, and many ovules are attached to axile placentae. The entire family is characterized by secondary pollen presentation in which protandry is combined with a close association of anthers around the style and introrse pollen discharge onto the style for presentation to pollinators. This syndrome is similar to that found in Lobeliaceae and Asteraceae, but invaginating stylar hairs are unique to the Campanulaceae.

Capsule characters vary considerably and provide the basis for most intrafamilial classification schemes. Campanuleae typically include taxa with capsules dehiscing by lateral pores, whereas Wahlenbergieae usually include taxa with capsules dehiscing by apical valves. Ovary characters, such as carpel number and position, have also been important in traditional classifications. For example, the monotypic tribe Platycodoneae [6] or subtribe Platycodinae (Table 1) is sometimes segregated. It is defined by carpels that are equal in number to and alternate with the calyx lobes, whereas in Campanuleae and Wahlenbergieae the carpels are often fewer than the calyx lobes, or if the same in number then opposite them [1,7,8]. Little correlation appears to exist among diagnostic features; therefore there is considerable taxonomic disagreement among classifications. In certain instances it is difficult to discern the rationale behind tribal placement of individual genera.

The high level of disagreement among both inter- and intrafamilial classifications of the Campanulaceae indicates that phylogenetic assessment of the family is needed. Cosner, in her thesis [9], included an early version of a portion of the work described here, and Eddie, in his thesis [10] developed phylogenetic hypotheses based on ITS sequence data and morphology. An expanded version of the ITS work has been published [11] but leaves some major lineages unsampled and the relationships among some major groups are unresolved or poorly supported. Further phylogenetic work is clearly warranted. The chloroplast genome has proven to be a useful tool for phylogenetic reconstruction. Chloroplast DNA (cpDNA) of land plants is highly conserved in nucleotide sequence as well as gene content and order; its relatively slow rate of evolution makes it an excellent molecule for phylogenetic and evolutionary studies [12]. Chloroplast genomes of photosynthetic angiosperms average about 160 kilobase pairs (kb) in size; the circular chromosome is divided by two copies of a large (in angiosperms usually about 25 kb) inverted repeat (IR) into large and small single copy regions (LSC and SSC, respectively) [13,14]. Restriction site mapping, gene sequencing, and analysis of gene order rearrangements have been used to study cpDNA variation for phylogenetic investigations [12]. Here we use the distribution of gene order changes in the chloroplast genomes of the Campanulaceae to estimate phylogenetic relationships in the family.

Generally, major gene order changes are rare. Therefore, when they occur, such mutations are extremely useful as phylogenetic markers because they are readily polarized and typically lack homoplasy [15-17]. Four categories of cpDNA gene order rearrangements have been proposed: 1) inversions, 2) insertions or deletions, 3) IR expansion or contraction or loss, and 4) transpositions; all of which may have occurred during chloroplast genome evolution in the Campanulaceae [18]. When rearrangements have been discovered elsewhere, they are generally few and easily characterized. The distributions of such characters make effective markers of monophyletic groups. For example, both the loss of one copy of the IR and inversions are extremely useful characters in legume phylogeny [19,20], defining large clades within the family. Other examples of phylogenetically informative inversions are found within Asteraceae [21], Ranunculaceae [22,23], ferns [24,25], and vascular plants [26]. Many other examples could be cited.

The earlier work of Cosner [9,18] and Knox [27,28] characterized some chloroplast genomes of the Campanulales and identified a number of rearrangements relative to the consensus gene order of angiosperms found in tobacco. Members of the Lobeliaceae exhibit multiple rearrangements but are less rearranged than the Campanulaceae. Three rearrangements may be shared between the two families – a loss of the *accD *gene, the expansion of the inverted repeat into the small single copy region, and, perhaps, an inversion of the region corresponding to tobacco probes 40–44. Then, within the Campanulaceae, more than 40 inversions, more than eight putative transpositions, two additional gene losses, additional IR expansion or contraction events and 18 large insertions greater than 5 kb in size may have contributed to observed differences among the chloroplast genomes sampled [9]. Due to this unprecedented number of gene order mutations, it is not possible to unambiguously determine the evolutionary order of most events or in some cases to even define the events themselves. This complex situation poses special problems for using these rearrangements to estimate phylogenetic relationships. In this paper we develop alternative character codings for the data and compare the results of parsimony analyses of the different data sets. In addition, we compare the ability of the gene order data to support robust phylogenetic hypotheses to that of sequence data from *rbcL *and ITS. Finally, the phylogenetic implications of the cpDNA rearrangement data for the Campanulaceae are discussed.

## Results

**Figure 1:**
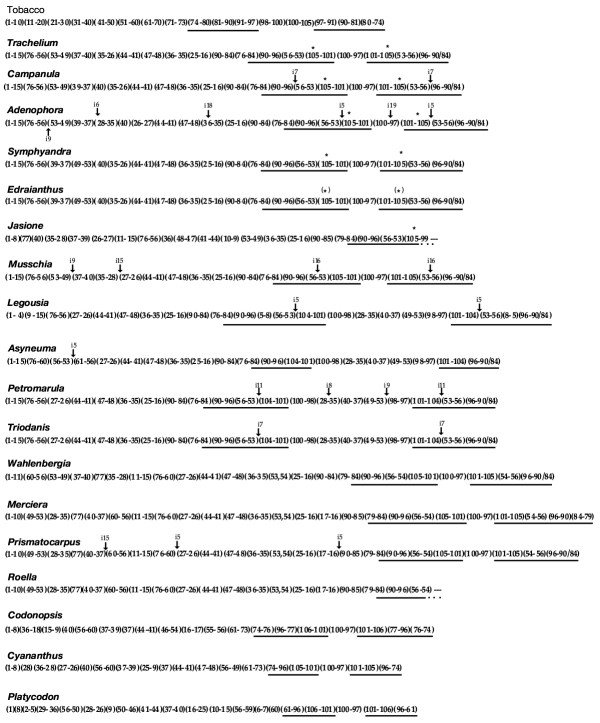
Linearized cpDNA maps for 18 species (Table 2) of Campanulaceae (plus tobacco) showing order in which the consecutively numbered tobacco probes hybridized. Lines under maps indicate location and extent of IR. Asterisks indicate the position of the putative *23S rDNA *duplicative transposition; parenthetical asterisk (*) indicates partial deletion/divergence of the *23S rDNA *transposition. Size and location of large insertions designated by "i" followed by size in kb (insertions less than 5 kb not shown).

Our data indicate that the eighteen mapped Campanulaceae chloroplast genomes (Table 2) are drastically rearranged relative to those of other land plants (Fig. 1). The tobacco cpDNA gene order represents the consensus gene order for angiosperms [13,15]. Therefore rearrangements in Campanulaceae chloroplast genomes were identified relative to tobacco. Because characterizing specific mutational events was not always possible three different coding methods (Matrix 1, 2 and 3) were developed. Matrix 1 coded all gene order changes as endpoints (derived adjacencies, relative to tobacco, were identified and scored for presence/absence). Matrix 2 and 3 involved recoding some endpoint characters to recognize 31 specific mutations. Matrix 2 and 3 were analyzed with and without weighting. See Methods for additional details on character encoding and analyses.

**Table 2:** Species of Campanulaceae mapped for chloroplast DNA structural rearrangments.

**Species**	**Source**	**Voucher**^a^
*Adenophora confusa *Nannf.	R.C. Haberle 179	TEX
*Asyneuma virgatum *(Labill.) Bourm.	Berlin-Dahlem^b ^0104	
*Campanula elatines *L.	T. Ayers 88–287	BH
*Codonopsis viridis *Wall.	T. Ayers 88–229	BH
*Cyananthus lobatus *Wall. ex Benth.	M. Cosner 179	OS
*Edraianthus graminifolius *(L.) A.DC.	T. Ayers 88–195	BH
*Jasione heldreichii *Boiss. & Orph.	T. Ayers 88–208	BH
*Legousia falcata *(Ten.) Fritsch ex Janch.	Berlin-Dahlem^b ^0143	
*Merciera tenuifolia *(L.f.) A. DC.	K. Steiner 2445	OS
*Musschia aurea *Dumort	T. Ayers 88–274	BH
*Petromarula pinnata *(L.) A. DC.	T. Ayers s.n.^c^	BH
*Platycodon grandiflorus *(Jacq.) A. DC.	T. Ayers 88–216	BH
*Prismatocarpus diffusus *(L.f.) A. DC.	K. Steiner 2448	OS
*Roella ciliata *L.	T. Ayers s.n.^c^	BH
*Symphyandra hofmannii *Pant.	T. Ayers 88–225	BH
*Trachelium caeruleum *L.	M. Cosner 173	OS
*Triodanis perfoliata *(L.) Nieuwl.	M. Cosner 178	OS
*Wahlenbergia gloriosa *Lothian	T. Ayers 88–217	OS

a, abbreviations for herbaria: BH = Bailey Hortorium (Cornell University, Ithaca, NY); OS = Ohio State University Herbarium (Columbus); TEX = University of Texas Herbarium (Austin)

b, Botanischer Garten and Botanisches Museum, Berlin-Dahlem

c, s.n. = *sin numero *(no number assigned by collector)

**Figure 2:**
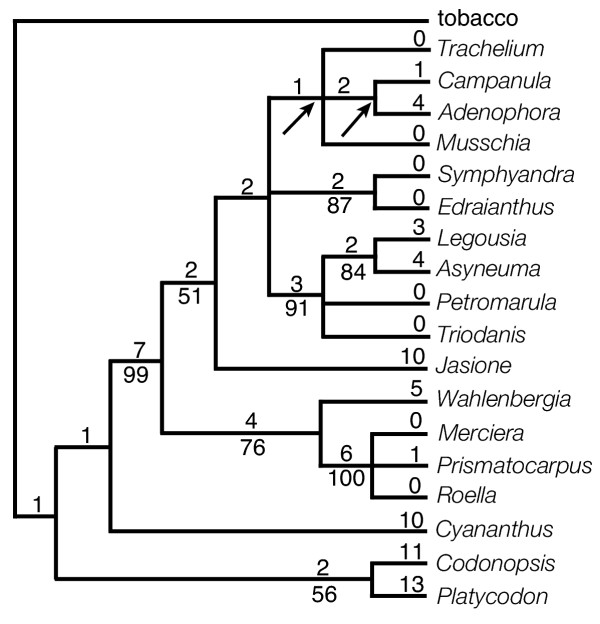
One of six shortest trees obtained in the maximum parsimony analysis of Matrix 1. Tree length is 97 steps; consistency index is 0.81 (with autapomorphies, 0.66 without). The number of character changes is given above the branches and bootstrap values (where greater than 50) are given below. Arrows indicate nodes that collapse in the strict consensus of all six shortest trees.

Seventy-nine variable characters were included in the endpoints only matrix (Matrix 1). Forty-two of the derived character states were unique to a single taxon and 37 were phylogenetically-informative. Six trees of 97 steps were obtained with consistency indices of 0.81 with autapomorphies included and 0.67 with autapomorphies excluded (CI = 0.81/0.67). Ten nodes were common to the six shortest trees (Fig. 2). Eight of those ten nodes have bootstrap values (BS) greater than 50, but BS exceeded 90 for only three nodes.

To construct Matrix 2 and Matrix 3, we interpreted endpoints as events where possible. Under our interpretation, several types of rearrangements contributed to cpDNA evolution in the family, including multiple inversions (scored primarily as endpoints), five IR expansion or contraction events, eight transpositions, two deletions, and 14 large insertions greater than 5 kb in size (Table 3). Although transposition probably does occur, at least occasionally, in the chloroplast genome [29], it is not a common mechanism of rearrangement. Still, in some instances transposition could explain rearranged gene orders with fewer steps than multiple inversions and so we hypothesized transposition events in some cases. Matrix 2 and 3 each were composed of 84 variable characters of which thirty-one and thirty-four, respectively, were parsimony informative.

**Table 3:** List of chloroplast DNA rearrangement characters. Numbers refer to tobacco cpDNA hybridization probes. Endpoints are given as novel probe adjacencies. Other rearrangement types are indicated as: T = transposition (T' = secondary transposition [14] of most of 53–56); I = inversion; i = insertion; D = deletion (or divergence); IRc and IRe = IR contraction or expansion, respectively (followed by single copy region affected). Characters marked with asterisks (*) are those rescored in Matrix 3 relative to Matrix 2.

*1. 11/60	22. T (53,54)	43. 40/56	64. i (15)
*2. 56/53	23. T (53–56)	44. 39/37	65. IR^e ^(LSC)
*3. 49/37	24. 98/28	45. 37/44	66. T (5–8)
4. 40/35	25. 53/98	46. 56/61	67. T (6–9)
*5. 28/11	26. T' (53–56)	47. 76/96	68. i (9)
6. 15/76	27. 49/39	48. 77/106	69. i (18)
7. 60/27	*28. 37/40	49. i (5)	70. l (60–61)
*8. 26/44	29. 56/39	50. 39/25	71. T (28)
9. 41/47	30. 53/40	51. 9/37	72. T (16–17)
10. 48/36	31. 37/28	52. 48/56	73. i 7
11. 35/25	32. 40/26	53. 49/61	74. IR^e ^(SSC)
12. 16/90	33. 27/44	54. 5/29	75. i (8)
13. T (93)	34. 8/40	55. 50/28	76. i (9)
14. 84/76	35. 39/26	56. 26/50	77. i (9)
15. 84/90	36. 27/11	57. 40/16	78. i (15)
16. 10/49	37. 56/36	58. 25/10	79. i (16)
17. 53/28	38. 44/10	59. 15/56	80. D (93)
18. 37/60	39. 9/53	60. IRc (LSC)	81. i (5)
19. 56/11	40. 49/36	61. IR^e ^(SSC)	82. i (6)
20. I (16–17)	41. 8/36	62. IR^e ^(LSC)	83. i (19)
21. 56/27	42. 9/40	63. D (45–46)	84. i (5)

**Figure 3:**
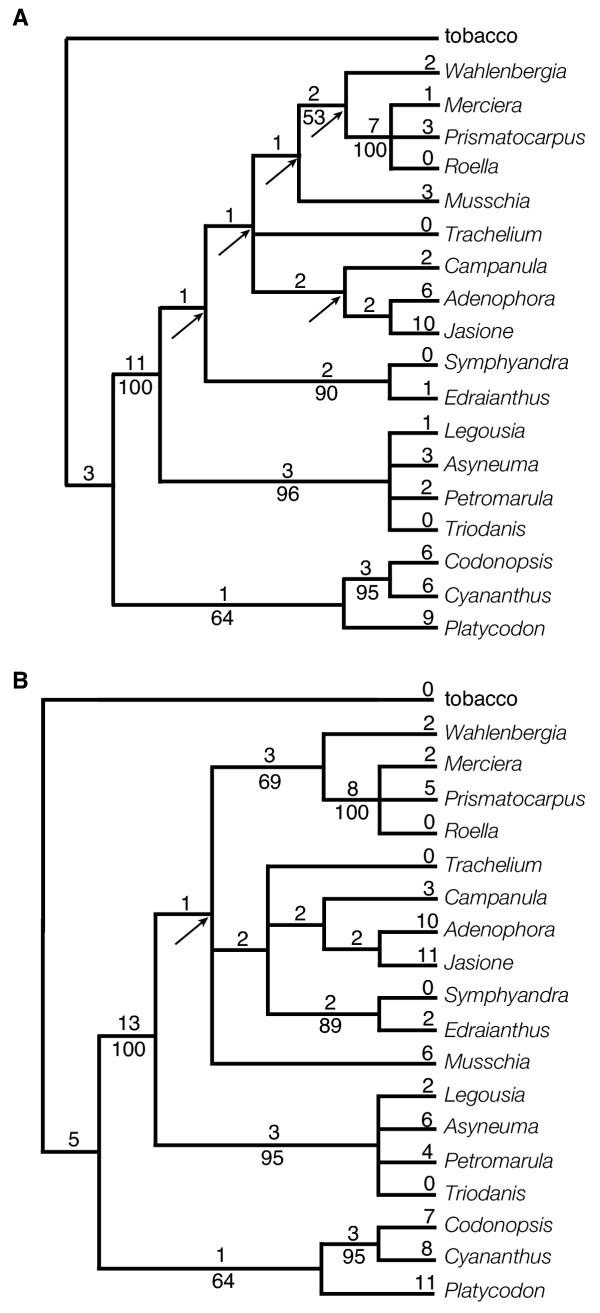
Trees obtained in unweighted (a) and weighted analyses (b) of Matrix 2. Fig. 3a shows one of 241 shortest trees of 93 steps; consistency index is 0.90 (including autapomorphies, 0.78 without). Fig. 3b shows one of 12 shortest trees of 125 steps; consistency index is 0.93 (including autapomorphies, 0.80 without). The number of character changes is given above the branches and bootstrap values are given below. Arrows indicate nodes that collapse in the strict consensus of all the shortest trees.

**Figure 4:**
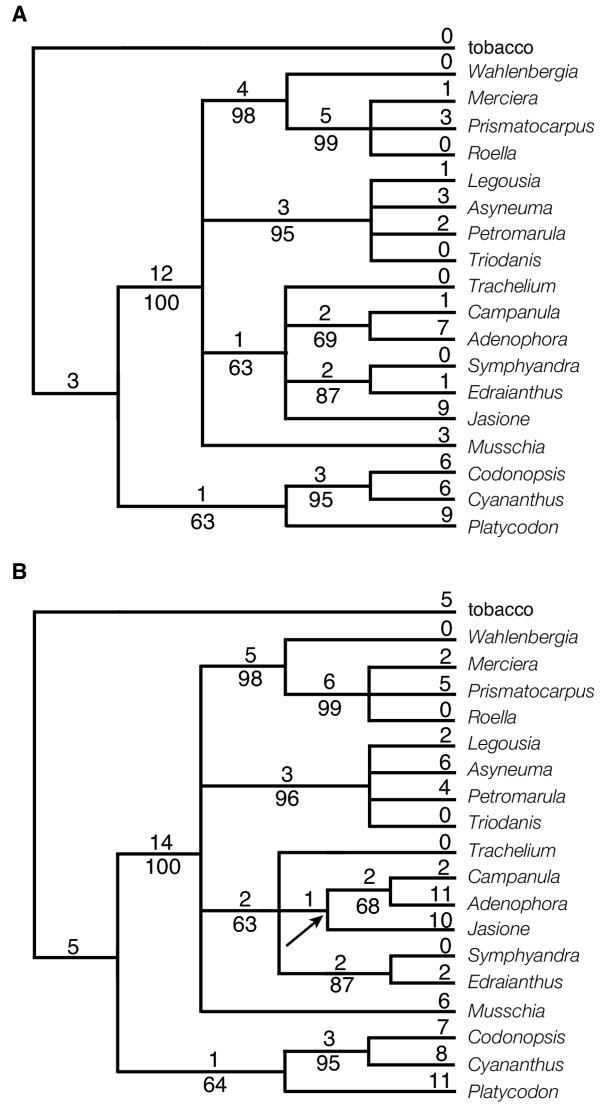
The two shortest trees obtained in both the unweighted and weighted analyses of Matrix 3. The trees are 87 steps long without weights and 125 steps when weights are applied. The consistency index of the trees in the unweighted analysis is 0.97 (including autapomorphies, 0.92 without); in the analysis with weights applied the CI is 0.97 (including autapomorphies, 0.93 without). Values given on the upper tree (Fig. 4a) pertain to the unweighted analysis, values on the lower tree (Fig. 4b) to the weighted analysis. The number of character changes is given above the branches and bootstrap values are given below. Arrows indicate nodes that collapse in the strict consensus of the shortest trees.

**Figure 5:**
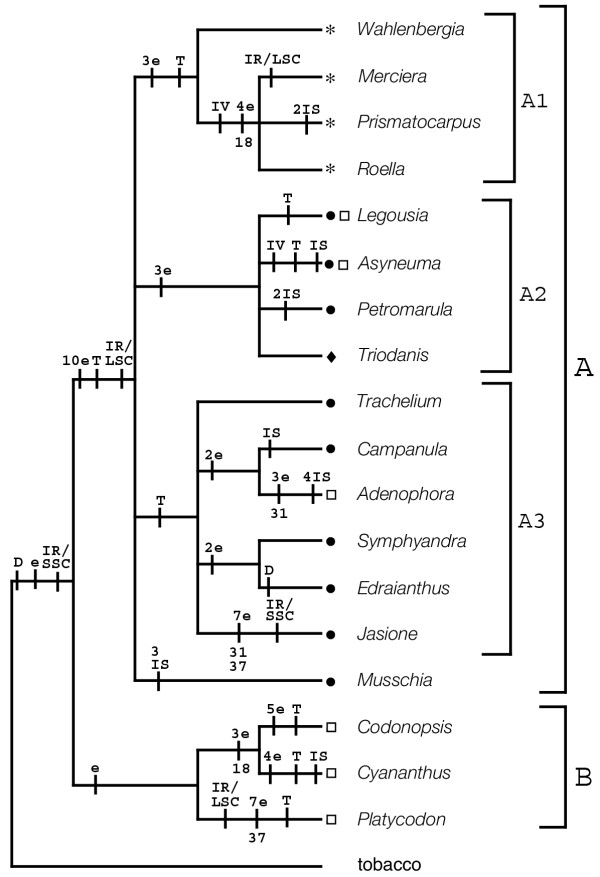
Strict consensus tree of the two equally parsimonious trees from the analysis of Matrix 3 (Fig. 4) showing character changes. Number and type of each change are indicated by e = endpoint, IV = inversion, IS = insertion >5 kb, T = transposition, and D = deletion/divergence. Three of the 84 characters (18, 31 and 37) exhibit homoplasy; each character is an endpoint that changes twice within the tree. Homoplastic changes are shown by the character number below the branch upon which the change occurs. To the right of the tree, brackets and letter/number designations indicate major clades discussed in the text. Symbols at the ends of the terminal branches indicate the geographic distribution of the taxa: □ = eastern Asia; ● = Europe (includes North Africa); ◆ = Americas (primarily North America); * = Southern Hemisphere (mainly Southern Africa).

The unweighted analysis of Matrix 2 produced 241 equally parsimonious trees of 93 steps (CI = 0.90/0.79). The strict consensus of the 241 trees includes six resolved nodes (Fig. 3a) all six of which were supported by BS values of at least 50 and five nodes were supported at 90% or above. The weighted analysis of Matrix 2 (Fig. 3b) resulted in 12 equally parsimonious trees of 125 steps (CI = 0.93/0.82). The strict consensus of the twelve trees retains ten resolved nodes. Seven of the ten nodes have BS values over 50 and for five nodes BS ≥ 90. Both analyses of Matrix 3 generated the same two equally parsimonious trees (Fig. 4). The lengths of the two trees were 87 steps (CI = 0.97/0.92) or 118 steps (CI = 0.97/0.93) depending on whether unweighted (Fig. 4a) or weighted (Fig. 4b) analyses were conducted. Only three endpoint characters are homoplasious in the Matrix 3 analyses (Fig. 5). The strict consensus of these two trees retains nine resolved nodes, all nine of which are supported with BS ≥ 50. Six (or five in the weighted analysis) nodes received strong support (BS ≥ 90).

All results (Figs. 2,3,4,5) indicate that *Codonopsis*, *Platycodon*, and *Cyananthus *are basal within the family. Analyses on Matrix 2 and 3 support a *Codonopsis *+ *Cyananthus *sister group relationship and a monophyletic basal clade whereas the Matrix 1 analysis supports a *Codonopsis *+ *Platycodon *sister group and a paraphyletic basal grade. Neither outcome is very well supported; the alternative scenarios each require only a single additional step in the other data set. Within the fifteen derived taxa some of the relationships are not resolved or resolved but weakly supported. However, some groupings are well supported in all analyses. The South African taxa, *Merciera*, *Prismatocarpus *and *Roella*, form a clade (BS = bootstrap value = 98 - 100). *Wahlenbergia *is the sister to these three taxa in all analyses with varying levels of support (BS = 82, 60, 69, 99, 99, in the five analyses based on gene order changes). Other groupings include a *Symphyandra *+ *Edraianthus *clade (BS = 86-91) and *Legousia *+ *Asyneuma *+ *Petromarula *+ *Triodanis *(BS = 94-100).

The five analyses had somewhat different characteristics (Table 4). For example, Matrix 1 and Matrix 3 analyses generated fewer equally-parsimonious trees than Matrix 2. The Matrix 1 analyses resolved the most nodes. Matrix 3 analyses exhibited the lowest amounts of homoplasy and supported the highest number of nodes BS ≥ 90. Comparing all results, no nodes with high bootstrap values (BS ≥ 90) were conflicted by other nodes of equally high value. However, there were three instances of incongruence involving nodes of lesser support – Matrix 1 and 3 analyses supported *Campanula *+ *Adenophora*, whereas Matrix 2 supported *Adenophora *+ *Jasione*; Matrix 1 supported *Codonopsis *+ *Platycodon *(BS = 50), whereas Matrix 2 and 3 supported *Codonopsis *+ *Cyananthus *(BS = 94-99); and Matrix 1 supported (weakly, BS = 57) the placement of *Cyananthus *at the base of the derived clade, whereas Matrix 2 and 3 analyses supported the monophyly of the basal group (BS = 56-68). One clade, *Legousia *+ *Asyneuma *(BS = 87), was recovered only by the Matrix 1 analysis within a clade not further resolved by the other gene order analyses.

**Table 4:** Comparison of characteristics from the different analyses, best values for each characteristic shown in bold.

	Matrix 1	Matrix 2, no weights	Matrix 2 weighted	Matrix 3, no weights	Matrix 3 weighted	*rbcL*	ITS*
Number of MP trees	6	241	12	2	2	9	**1**
Number of resolved nodes in consensus of MP trees	10	6	10	9	9	**14**	13
Nodes retained in consensus of all trees to 1% longer	6	5	5	6	**7**	5	2
CI (with / without autapomorphies)	0.81/0.67	0.90/0.79	0.93/0.82	0.97/0.93	**0.97/0.94**	0.77/0.66	0.69/0.60
Number of nodes BS ≥ 50	8	7	7	9	9	**13**	10
Number of nodes BS ≥ 90	3	5	4	**6**	5	5	**6**
Average bootstrap value of resolved nodes	72	**91**	72	85	86	78	74
Number characters (PI)	37	31		34		116	**195**
Total homoplastic characters (number/percent of PI)	15/40.5%	8/25.8%		**3/8.8%**		63/54.3%	141/71.9%
Homoplastic characters with one excess change	12/32.4%	7/22.6%		**3/8.8%**		48/41.4%	82/41.8%
Homoplastic characters with two excess changes	3/8.1%	1/3.2%		**0**		14/12.1%	41/20.9%
Homoplastic characters with three or more excess changes	**0**	**0**		**0**		1/0.1%	18/9.2%

*the ITS analysis includes 3 fewer taxa than the others

**Figure 6:**
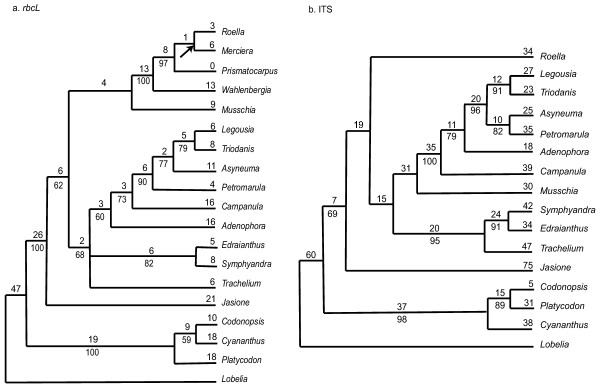
Trees obtained from sequence data. The values below nodes are bootstrap percentages; the values above the nodes indicate the number of changes that occur on that branch. Fig. 6a. The one shortest tree (716 steps) based on ITS sequence data. The CI is 0.69 with autapomorphies and 0.60 without. Fig. 6b. One of the nine shortest trees (338 steps) based on *rbcL *sequence data, CI = 0.77/0.66. The node that collapses in the strict consensus of the nine trees is marked with an arrow.

We included sequence data here mainly to allow for a comparison with gene order data in terms of phylogenetic utility. The *rbcL *data from the same eighteen taxa (Table 5) provided 116 parsimony-informative characters that, when analyzed, yielded nine shortest equally-parsimonious trees of 338 steps (C = 0.77/0.66). The strict consensus of the nine trees retained fourteen nodes (fig. 6a), thirteen of which had BS ≥ 50 and four of which were supported BS ≥ 90. The ITS data of Eddie et al [11] from taxa equivalent to fifteen of the eighteen mapped taxa (Table 5) provided 196 parsimony-informative characters from which a single most parsimonious tree of 716 steps (fig. 6b) was generated (CI = 0.69/0.60). The tree contains thirteen resolved nodes of which ten had BS ≥ 50 and four had BS ≥ 90. The two sequence data sets had lower CI values than any of the gene order analyses and a higher percentage of homoplastic characters (Table 4). The ITS data had especially high levels of homoplasy; the ITS data had a higher percentage of characters that change three or more times in excess than the Matrix 3 analyses had for total homoplastic characters (Table 4). In the Matrix 3 analysis only three characters (endpoints) are required to change more than once over the most parsimonious tree; each has one excess change. With the inclusion of the sequence-based analyses, there were additional instances of incongruence between weakly supported nodes: 1) The placement of *Musschia *and *Jasione *varies between the *rbcL *and ITS results (the placement of these taxa is largely unresolved by the gene order data); 2) In both sequence-based trees, *Campanula *and *Adenophora *are separate lineages (rather than sister taxa) basal to the *Legousia*-*Asyneuma*-*Triodanis*-*Petromarula *clade, whereas in the gene order analyses they are allied to *Symphyandra*-*Edraianthus*, and *Trachelium*; 3) Matrix 1 supports a *Legousia*-*Asyneuma *clade, whereas a *Legousia*-*Triodanis *clade occurs in the sequence-based trees; and 4) Matrix 1 and ITS support a *Codonopsis*-*Platycodon *grouping within the basal clade, whereas *rbcL *and Matrix 2 and 3 analyses support *Codonopsis*-*Cyananathus. *Among these instances of disagreement between weakly supported nodes, there is no general pattern of disagreement between the sequence data and the gene order analyses. And among strongly supported nodes, again, there is complete agreement, among all analyses-sequence and gene order.

**Table 5:** Taxa for which *rbcL *and ITS data were analyzed.

**Gene Order/rbcL Species**	**GenBank Accession*rbcL***	**ITS "equivalent" taxon**	**GenBank Accession ITS [11]**
*Lobelia cardinalis*	AY655144	*Lobelia tenera*	AF054938
*Adenophora confusa*	AY655145	*Adenophora divaricata1*	AY322005 & AY331418
*Asyneuma virgatum*	AY655146	*Asyneuma japonica*	AF183437 & AF18343
*Campanula elatines*	AY655147	*Campanula lusitanica*	AY322025 & AY331438
*Codonopsis viridis*	AY655148	*Codonopsis lanceolata*	AY322048 & AY331461
*Cyananthus lobatus*	L18795 [40]	*Cyananthus lobatus*	AY322050 & AY331463
*Edraianthus*	AY655150	*Edraianthus*	AY322052 & AY331465
*graminifolius*		*graminifolius*	
*Jasione heldreichii*	AY655151	*Jasione crispa*	AY322059 & AY331472
*Legousia falcata*	AY655151	*Legousia speculum-*	AY322065 & AY331478
		*veneris*	
*Merciera tenuifolia*	AY655153	No equivalent	NA
*Musschia aurea*	AY655154	*Musschia aurea*	AY322067 & AY331481
*Petromarula pinnata*	AY655155	*Petromarula pinnata*	AY322069 & AY331482
*Platycodon grandiflorus*	AY655156	*Platycodon grandiflorus*	AY322074 & AY331487
*Prismatocarpus diffusus*	AY655157	No equivalent	NA
*Roella ciliata*	AY655158	*Roella ciliata*	AY322074 & AY331487
*Symphyandra hofmanni*	AY655159	*Symphyandra hofmanni*	AY322076 & AY331489
*Trachelium caeruleum*	L18793 40	*Trachelium caeruleum*	AY322078 & AY331491
*Triodanis perfoliata*	AY655160	*Triodanis leptocarpa*	AY322079 & AY331492
*Wahlenbergia gloriosa*	AY655161	No equivalent	NA

## Discussion

### Phylogenetic analysis of cpDNA rearrangements

The relatively large number of gene order mutations that have occurred in the Campanulaceae chloroplast genomes causes difficulties when interpreting their phylogenetic significance. The phylogenetic analysis of such a complex set of cpDNA rearrangements within a group of plants is without precedent. The first problem was simply defining individual mutational events. Although the ideal way to analyze rearrangement data is to determine presence or absence of specific events, in the Campanulaceae, this was not possible in many cases given our present knowledge. Where multiple overlapping rearrangements have occurred between genomes, the two specific endpoints that define a particular inversion may not be determinable. Because of the inherent complexity of the data, we felt a new method of character analysis of the rearrangement data was warranted. Our approach involved coding endpoints, along with more easily defined rearrangements, as characters for different cladistic analyses. Endpoints were defined as two non-contiguous tobacco regions that are now adjacent in the genomes of one or more species.

Using endpoints as characters is advantageous. It allows for the incorporation into the analysis of data that could not be used if only unambiguously interpreted events were included. However, using endpoints as characters has several drawbacks, including the inadvertent weighting of certain events over others. Inversions necessarily produce two endpoints, and transpositions three, whereas gene losses and IR boundary changes produce a single endpoint. Therefore inversions would be included twice and transpositions three times if scored as "independent" endpoints rather than events. Plus, if both endpoints of an inversion are still intact in a genome, the inversion is scored twice, if only a single endpoint remains the inversion is counted only once, and if both endpoints have been lost (through further mutation) the inversion will not be included at all. This may represent a problem in the Campanulaceae analyses because there appears to be a mixture of event types and at least some endpoint reuse [18].

Our inclusion of transposition as a possible mechanism for gene order mutation in the Campanulaceae chloroplast genomes is problematic. Definitive evidence supporting the occurrence of transposition in the plastid genome is lacking. Transposition has been invoked to explain chloroplast DNA rearrangements, for example in "subclover" [30] and wheat [31,32]. In these cases, transposition has been supported using parsimony arguments (one transposition explaining a change with fewer steps than three inversions) or using the existence of inverted- and direct-repeat sequence motifs near the boundaries of rearrangements [33]. In Campanulaceae, some lines of evidence in addition to parsimony suggest the possibility of transposition as a mechanism. First, the abundance of rearrangement events within the family suggests some mechanism that facilitates gene order mutation; transposition is one such process. Second, the segment of the genome defined by tobacco probes 53–56 is now located, in most of the derived taxa, within the inverted repeat. The region from which it has been removed appears otherwise undisturbed. In *Asyneuma*, the 53–56 region has been secondarily removed from the IR and returned to near its original location leaving behind small portions of 53 and 56 in the IR, detectable using southern hybridization. In *Wahlenbergia*, *Merciera*, *Prismatocarpus *and *Roella*, the 53, 54 portion of the 53–56 block has moved from the IR back to the LSC. One explanation for the high level of rearrangement apparently associated with this segment is that the region contains a transposable element. Third, a possible duplicative transposition is suggested (Fig. 1) in *Trachelium *[18] and five other taxa [9]. In addition to a full-length (presumably functional) copy of the 23S rRNA gene, a partial copy is located within *ycf1*. Transposition is one manner in which segments of DNA can be both copied and moved within a genome. None of our data are definitive. The observed rearrangements could have taken place as the result of multiple inversions. Therefore, it is important to note that if transposition is not active in the Campanulaceae genome, our phylogenetic results will not be greatly affected. Events coded in Matrix 2 and 3 as single transpositions would be underweighted inversions if incorrectly interpreted. The fact that the analysis of Matrix 1 yields results compatible with those of the matrices that include transpositions suggests that, if our interpretation is erroneous, it does not affect the phylogenetic conclusions.

Our three methods of character scoring did yield largely compatible results in our analyses. Relationships that were strongly supported in one analysis were found in all analyses. Events make more desirable characters but they will only improve analyses if the postulated events are the correct ones. Comparing analyses that include event interpretations with endpoint only analyses is one way to determine the phylogenetic effects of the hypotheses of events used. Endpoint only analyses also allow studies that minimize a priori assumptions about the evolutionary events. It is possible that more complex evolutionary scenarios occurred, in which some inversions evolved in parallel, or in which similar gene orders resulted from a different set of inversions. The parsimony analyses may underestimate the number of inversions shared between primitive and advanced genera, because evidence of shared inversions may have been lost. Although we have attempted to produce the simplest evolutionary schemes, it is very possible that longer, more complicated scenarios actually occurred, especially given that the Campanulaceae seem predisposed to cpDNA rearrangements. However, given the congruence of the results among our various analyses, we feel our phylogeny is a reasonable estimate of relationships within the family. Elsewhere, we have analyzed a reduced subset of characters and taxa for the Campanulaceae cpDNA data set using endpoint scoring and constructing trees using breakpoint distances among other methods (e.g., [34]). Other computational biologists have also used this reduced data matrix to test different methods of phylogeny reconstruction based on gene order data (e.g., [35,36]). These various studies produced trees that are largely congruent with those generated in this paper suggesting that the Campanulaceae cpDNA gene order data are providing a consistent estimate of phylogenetic relationships given any logical method of scoring and analysis. Although the rearrangements in Campanulaceae are complex, the phylogenetic utility of the gene order data is evident. In most previous examples of phylogenetic use of rearrangements the small number of events allowed for the circumscription of only very broad groups [15]. Because there are so many rearrangements in the Campanulaceae, smaller groups can be identified. This has resulted in the most highly resolved phylogeny as yet developed based entirely on cpDNA rearrangements.

Not only do these data support a well-resolved phylogeny but they provide robust support of several nodes. Matrix 3 supports as many nodes at BS ≥ 90 as ITS and more than *rbcL*. In matrix 3, only three endpoint characters (8.8% of parsimony-informative characters) are homoplastic, each changing one extra time over the tree. In contrast, within ITS, 18 characters (9.2% of parsimony-informative characters) change three or more extra times over the tree, and 71.9% of characters are homoplastic. Presumably because of this high level of homoplasy, only two nodes are retained in the consensus of all ITS trees from the shortest to 1% longer, whereas seven nodes are retained in matrix 3 trees "to 1% longer" – the highest number of any of the analyses. Matrix 3, the matrix in which characters are most interpreted as mutational events, is especially strong in its performance, exceeding both sequence data sets in average bootstrap value per resolved node and CI (in addition to those characteristics just discussed). This suggests that the closer we can get to scoring the actual mutations the stronger gene order data will perform. Although the endpoint only matrix provides useful insights on relationships, we would argue that the extent to which these gene order characters cannot recover the phylogeny is directly related to our ability to define individual mutational events.

### Phylogenetic implications of the rearrangement data

Most traditional classifications of the Campanulaceae are based mainly on capsule dehiscence and ovary position and arrangement. As Kovanda [1] and Thulin [8] recognized, classification of the Campanulaceae based on capsule characters alone brings together otherwise radically different taxa. Neither the Campanuleae nor Wahlenbergieae (at whatever taxonomic rank) are monophyletic based on cpDNA rearrangements (Fig. 5). Likewise, no traditional classification (Table 1) suggests that *Codonopsis*, *Platycodon*, and *Cyananthus *are basal in the family as supported by both gene order and sequence data. Takhtajan's system [2] is something of an exception among traditional classifications; however, he suggested only *Cyananthus *(in its own tribe Cyanantheae) as the most primitive member of the family, placing *Platycodon *and *Codonopsis *in other tribes (Table 1).

In contrast, studies of pollen ultrastructure have indicated that *Platycodon*, *Codonopsis*, and *Cyananthus *are basal members of Campanulaceae [37,38]. These taxa have colpate to colporate apertures, whereas the remaining family members (as surveyed here) have porate grains [37-41]. The evolutionary scheme based on pollen morphology presented by Dunbar [38] suggests that *Cyananthus *(colpate) and *Codonopsis *(colpate) are more closely related to each other than either is to *Platycodon *(colporate), which is also supported by the gene order tree (Clade B, Fig. 5). Thulin [8] believed that pollen morphology should constitute a key part of any modern reassessment of relationships in the Campanulaceae. He suggested that all taxa with elongated apertures should be removed from Campanuleae and Wahlenbergieae, and those with porate grains removed from Schönland's Platycodinae. Following the removal of colpate and colporate taxa, Campanuleae *sensu *Schönland are comprised of Northern Hemisphere genera, whereas Wahlenbergieae contain Southern Hemisphere taxa, with the exceptions of *Edraianthus *and *Jasione *(although *Jasione *occurs in North Africa as well as Europe). The gene order data indicate that the affinities of *Jasione *and *Edraianthus *lie with Northern Hemisphere species rather than with Wahlenbergieae. The gene order data also are compatible with other available nucleotide data in addition to those reported here [[10,11,42], L. Raubeson, A. Oestriech and R. Jansen, unpublished data], a morphology-based cladistic study [10] and are also largely congruent with a serological study of the Campanulaceae [43]. Although the gene order and serological studies differed somewhat in the taxa sampled, both included a group containing *Trachelium *and *Campanula*. They also agreed in the grouping of *Asyneuma *and *Petromarula*. The only discrepancy was in the placement of *Legousia*; the serological study placed this genus basal to all others surveyed [43].

The groups delimited by cpDNA rearrangements also exhibit geographical integrity. *Wahlenbergia *is primarily a Southern Hemisphere Old World genus [44]; *W. gloriosa*, mapped for this study, is Australian [44]. *Roella*, *Merciera*, and *Prismatocarpus *are all endemic to South Africa [45-47]. The nine genera in the *Trachelium *and *Legousia *clades are primarily European to Eurasian, although *Triodanis *is endemic to North America and *Campanula *has a few North American representatives [5,48-50]. *Musschia *is endemic to the island of Madeira [51].

There has been considerable debate regarding the relationships among the four centers of taxonomic diversity of the Campanulaceae: Asia, Europe (especially the Mediterranean), South Africa, and western North America. Bentham [52] hypothesized a northern origin for Campanulaceae but he did not specify a particular region. Takhtajan [2] suggested a basal position of the Asian genus *Cyananthus*. Studies of pollen ultrastructure indicated that the Asian genera *Codonopsis*, *Cyananthus*, and *Platycodon *are basal members of the Campanulaceae [37,38]. Recent studies of the Campanulales [42,53,54] indicate that the order consists of several families, including the Campanulaceae, Cyphiaceae, Cyphocarpaceae, Lobeliaceae, Nemacladaceae, and Stylidaceae. Several of these families are restricted to the Southern Hemisphere (all but Nemacladaceae from North America and Campanulaceae which is cosmopolitan), implying that the Southern Hemisphere may be the ancestral area for the Campanulaceae [54]. Phylogenies based on *rbcL *sequence data position the Campanulaceae sister to the North American family Nemacladaceae [42,54]. Our cpDNA phylogeny based on genome rearrangements (Fig. 5) provides strong support for the basal position of the three examined Asian platycodonoid genera, suggesting that the early radiation of the family may have occurred in Asia rather than Africa. The genera from the Southern Hemisphere (*Merciera*, *Prismatocarpus*, *Roella*, and *Wahlenbergia*) are in a much more derived position in the cpDNA tree.

In addition, the gene order data suggest affinities of several controversial genera (Fig. 5). Schönland [48] united *Musschia *and *Platycodon *as Platycodinae, clearly incompatible with both our results and pollen evidence. *Musschia *is placed in the derived clade (A), although its exact placement varies among all the analyses, including *rbcL *and ITS. De Candolle [3] was unsure of *Merciera*'s taxonomic position because its four basal ovules and single-seeded (by abortion) unilocular capsule [43] are unique in the Campanulaceae [55]. This genus was later recognized as a separate tribe, Merciereae [56], but is allied with other southern African genera in the cpDNA analysis (Fig. 5). Takhtajan [2] placed *Merciera *with *Wahlenbergia*, *Roella *and *Prismatocarpus *in his Wahlenbergieae but also included other genera forming a polyphyletic group according to our results.

*Adenophora *and *Symphyandra *have been segregated from *Campanula *based on the presence of a conspicuous tubular nectariferous disc and connate anthers, respectively. *Adenophora *and *Campanula *are sister taxa in the gene order analyses (except those based on Matrix 2) and *Adenophora'*s chloroplast genome is derived relative to *Campanula'*s (Fig. 5). Further sampling within *Adenophora *and the large genus *Campanula *will be necessary to determine if this is a general result. *Symphyandra *is more closely related to *Edraianthus *than *Campanula *but all are within the A3 Clade (Fig. 5). *Edraianthus *has traditionally been considered close to *Wahlenbergia *[3] but this is not supported by any of the results reported here or by morphological studies of Hilliard and Burtt [57].

Much controversy surrounds the taxonomy of the genera *Triodanis *and *Legousia*. In some treatments, both genera were included under the illegitimate name *Specularia *(e.g. [3,48]). McVaugh [58,59] and Fernald [60] disagreed regarding the circumscription of the genera; Fernald felt that *Triodanis *as a genus is very weak and should be merged with *Legousia*. McVaugh [58] argued that the two genera should either remain separate or both be subsumed into *Campanula*. In his system, both species studied here (*T. perfoliata *and *L. falcata*) belong to *Triodanis*. As expected, *Triodanis *and *Legousia *belong to the same cpDNA clade (A2), united by an unusual mutation that transferred a large segment of the large single copy (LSC) region to the SSC region [9]. However, *Legousia *has a putative transposition not found in *Triodanis*, whereas *Triodanis *has a unique large insertion [9].

## Conclusions

Despite the difficulties in interpreting such a complex set of rearrangements, the systematic utility of chloroplast DNA in the Campanulaceae is evident. Our results support the division of the family into two groups previously unrecognized in any taxonomic treatment. In addition, numerous groupings within the larger, derived clade are strongly supported. The data indicate that traditional classifications based on fruit and ovary characters are unnatural, and suggest affinities of several difficult genera. Additional sampling within large genera, such as *Campanula *and *Wahlenbergia*, will be necessary to fully elucidate relationships among chloroplast genomes. It is likely that intrafamilial relationships can be further resolved by including other genera in rearrangement analyses. Although homoplasy is not absent in our data, it is low and considerably lower than some sequence data such as ITS. Although any reasonable scoring method for the gene order data generates results that are largely compatible among the different analyses, the more that the gene order data can be interpreted as actual mutational events (and the presence or absence of those events used as characters) the stronger will be the results. Even in cases such as this, with high levels of gene order complexity, cpDNA gene order mutations make excellent phylogenetic markers.

## Methods

Total DNA was isolated from one species in each of 18 genera in the Campanulaceae (Table 2) according to the CTAB method of Doyle and Doyle [61]. DNAs were digested with the restriction endonucleases *Bam*HI, *Bgl*II, *Eco*RI, *Eco*RV, *Hind*III, and *Sst*I, and double digests were carried out using *Hind*III and the remaining five enzymes. Hybridization probes consisted of 106 small tobacco cpDNA probes (average size 1.2 kb) provided by J. Palmer [62]. Twenty-one cloned *Hind*III cpDNA fragments from *Trachelium caeruleum *of the Campanulaceae were also used as hybridization probes [18]. Complete single and double digest restriction site maps were constructed for 16 of the 18 taxa, and nearly complete maps were constructed for the remaining two taxa, *Jasione *and *Roella *[9]. It was not possible to map the small single copy (SSC) region of *Roella *because hybridization signals became increasingly weak in later rounds of hybridization. In *Jasione*, rearrangements involving the IR/SSC junction and SSC region prohibited the complete resolution of the map. The restriction site maps were then interpreted as linear "number" maps representing the hybridization patterns of 106 consecutively numbered tobacco cpDNA probes for the 18 taxa (Fig. 1).

**Figure 7:**
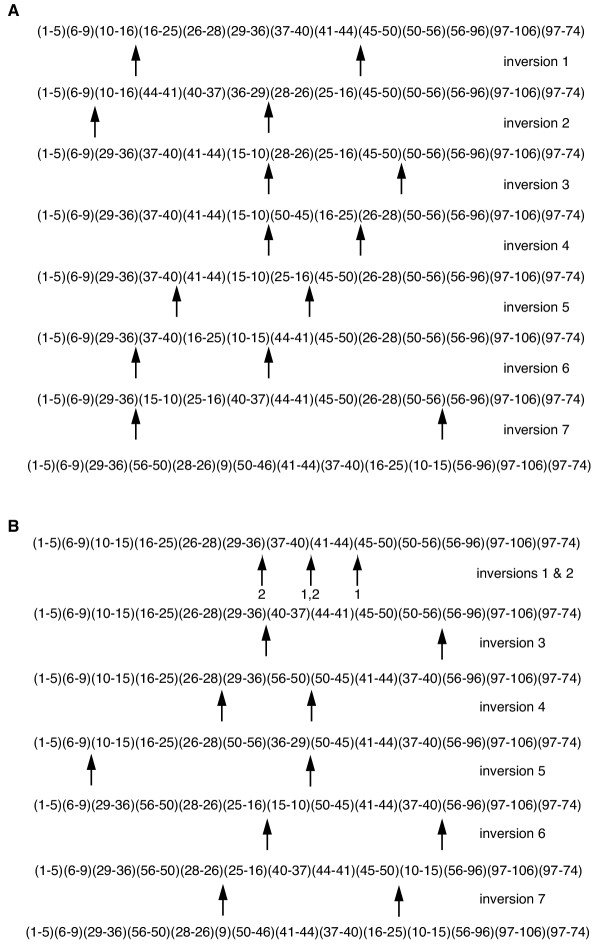
Two alternative models of seven inversions each to explain the evolution of *Platycodon *cpDNA structure from a tobacco-like ancestor. Numbers in parentheses show order of hybridized tobacco cpDNA probes. Inversion endpoints are shown by the arrows. Locations of regions represented by probes 6-7, 8, and 9 are believed to be the result of transposition [14]; these events are required in addition to the inversions to completely explain the new gene order.

Rearrangements were recognized as any change in the order of gene segments relative to the order observed in tobacco. The recognition of such disruptions is straightforward; the interpretation of the disruptions as actual mutational events can be quite complicated. As a hypothetical example, the ancestral order in a region may be 1-2-3-4-5-6; while the order 1-2-5-3-4-6 may be observed in a rearranged genome. In the rearranged genome 2-5, 5-3, and 4-6 are adjacencies that are derived relative to the ancestral order. But what set of events is responsible for the change? A simple transposition of 5 to the position between 2 and 3 can account for the difference in a single event. Alternatively, two inversions with one shared endpoint may be responsible or two inversions with unique endpoints followed by a transposition can explain the differences. Additional explanations would also be compatible with these data. On what basis do we choose among multiple scenarios? As an actual example, the chloroplast genome of *Platycodon *could have evolved from a tobacco-like ancestor by two different models each involving seven inversions (Fig. 7); not one inversion is common to the two scenarios. Thus in our initial approach to data analysis (generating Matrix 1) we did not define events, but utilized endpoints only. In the hypothetical example 2-5, 5-3, and 4-6 are "endpoints" -derived adjacencies absent in the ancestral gene order. Taxa with genomes that exhibit the derived adjacencies are coded as 1 for those characters and those with the ancestral condition as 0.

We constructed two additional matrices that did include events since some endpoints (or combination of endpoints) seemed readily interpretable. For example, if a region of the genome was simply reversed in order (i.e., 1-4-3-2-5 relative to 1-2-3-4-5) we assumed that an inversion had taken place to result in the different arrangements of gene segments. Likewise if genomes differed in content of the IR, we assumed that single duplication or loss events were responsible. Making such inferences, we constructed Matrix 2 that is composed of 31 events and 53 endpoints. We then went further and constructed hypotheses of rearrangement events to account for the differences among the genomes of the three major clades delimited among the fifteen derived taxa [9]. If these scenarios indicated that an inversion likely was shared between two or more genera, the taxa were coded as having an endpoint even if the endpoint has been lost due to disruption by subsequent events. We were conservative in our application of this approach and only six endpoint scorings were modified in Matrix 3 compared to Matrix 2. To summarize, we produced three data matrices that represented increasing levels of interpretation of endpoints as actual events.

Cladistic analyses were performed on each of the three data sets using equal weighting of all included characters. The second and third matrices were also analyzed giving weights of two to all non-endpoint characters. This weighting represents an attempt to compensate for the unintentional weight given to inversions in which both endpoints are present. This, of course, results in down-weighting inversions in which only one endpoint remains and fails to include inversions whose endpoints are both absent.

Finally, to allow for a direct comparison of performance between gene order data and sequence data over the same taxa, we conducted maximum parsimony analyses of ITS and *rbcL *data. The ITS sequences were generated and aligned by Eddie et al [11]. We determined taxa equal or equivalent to our taxa and performed analyses on just those taxa from the Eddie matrix (Table 5); only fifteen of our eighteen taxa were represented. We generated *rbcL *data to add to the two taxa already available [42] so that we had *rbcL *sequence data from all of the eighteen mapped taxa. Exactly the same DNAs were used.

In generating the *rbcL *sequences, we PCR-amplified about 1370 bp of the gene in 50 μl reactions containing: 1 μl unquantified total genomic DNA, 0.2 mM each dNTP, 2.5 mM MgCl2, 50 mM KCl, 10 mM Tris-HCl (pH 9.0), 0.4 μM each primer, and 1 unit Taq polymerase. Cycling conditions were as follows: 1 95°C denaturation step for 3 minutes 30 seconds, 30 cycles of 1 minute at 95°C, 1 minute at 55°C, and 1 minute 30 seconds at 72°C, and finally a 7 minute 72°C step. The PCR primers plus two internal primers were used for sequencing; the forward amplification primer and two internal primers were designed by G. Zurawski (his Z-1, Z-427 and Z-895). The Zurawski primer commonly used as the reverse amplification primer did not work in many Campanulaceae; we designed an alternative: 5'-GTATCCATTGCGCAAACTC-3'. For sequencing, two successful PCR reactions were combined and then cleaned (and concentrated) using the Qiagen QIAquick PCR Purification Kit (catalog number 28104). Depending on the concentration of the recovered product, 0.5–2 μl of this template was cycle sequenced and resolved on an ABI Prism 377 Automatic DNA Sequencer. Electropherograms were inspected, and then sequences were edited and assembled using Sequencher, vers 3.1 (Gene Codes Corp.) The sequences have been deposited in GenBank (accession numbers in Table 5). Alignment was performed by Sequencher and adjusted manually. Alignment of the *rbcL *sequences was very straightforward.

For all parsimony analyses, searches were conducted using the branch and bound algorithm in PAUP* 4.0b10 (PPC) [63]. Tobacco was used as the outgroup for the gene order data since it has the ancestral chloroplast genome gene order for the angiosperms [15,26] and *Lobelia *was used as the outgroup for the sequence data. See the additional file - data file 1 – for the Nexus file used in the PAUP analyses. This file includes the three gene order matrices and the *rbcL *alignment. The ITS alignment of Eddie et al [11] is available online [64]. The strength of the support, in each data set, for monophyletic groups was evaluated by calculating bootstrap values [65] using 10,000 heuristic (TBR, multrees option) replicates. In addition, for each matrix, analyses were performed to generate all trees from the shortest to one percent longer. We used a percentage rather than an equal number of steps in an attempt to make an equivalent comparison among the different sized data sets. A consensus of these trees was determined and the number of nodes retained "to 1% longer" was calculated.

## Authors' contribution

MEC performed the DNA isolations and Southern hybridizations, mapped the genomes, performed character codings and analyses for Matrix 2 and Matrix 3, and wrote up her work as a chapter of her Ph.D. thesis. LAR confirmed genome maps and character codings, updated MEC's analyses, added MATRIX 1 and its analysis, generated the *rbcL *sequence data, analysed ITS and *rbcL *data, and modified the thesis chapter for publication. RKJ assisted in all aspects of the work.

## Supplementary Material

Additional File 1 One additional file is provided – data file 1. This file is in NEXUS format and includes data for Matrix 1, Matrix 2, and Matrix 3 encodings and the *rbcL *data, concatenated for each taxon. PAUP statements in the file identify each individual data matrix and the characters in Matrix 2 and 3 that are weighted in some analyses. Click here for file
